# Evolutionary-Driven Convolutional Deep Belief Network for the Classification of Macular Edema in Retinal Fundus Images

**DOI:** 10.3390/jimaging11040123

**Published:** 2025-04-21

**Authors:** Rafael A. García-Ramírez, Ivan Cruz-Aceves, Arturo Hernández-Aguirre, Gloria P. Trujillo-Sánchez, Martha A. Hernandez-González

**Affiliations:** 1 Centro de Investigación en Matemáticas (CIMAT), Guanajuato 36023, Guanajuato, Mexico; rafael.ramirez@cimat.mx (R.A.G.-R.); artha@cimat.mx (A.H.-A.); 2 SECIHTI-Centro de Investigación en Matemáticas, Guanajuato 36023, Guanajuato, Mexico; 3 Unidad Medica de Alta Especialidad, Hospital de Especialidades No. 1 IMSS, León 37320, Guanajuato, Mexico; trujillosanchezgloriapaulina@gmail.com; 4 División de Ciencias de la Salud, Universidad de Guanajuato, Campus León, León 37544, Guanajuato, Mexico; ma.hernandezg@ugto.mx

**Keywords:** automatic classification, convolutional deep belief network, macular edema, hard exudates, microhemorrhages, genetic algorithm

## Abstract

Early detection of diabetic retinopathy is critical for preserving vision in diabetic patients. The classification of lesions in Retinal fundus images, particularly macular edema, is an essential diagnostic tool, yet it presents a significant learning curve for both novice and experienced ophthalmologists. To address this challenge, a novel Convolutional Deep Belief Network (CDBN) is proposed to classify image patches into three distinct categories: two types of macular edema—microhemorrhages and hard exudates—and a healthy category. The method leverages high-level feature extraction to mitigate issues arising from the high similarity of low-level features in noisy images. Additionally, a Real-Coded Genetic Algorithm optimizes the parameters of Gabor filters and the network, ensuring optimal feature extraction and classification performance. Experimental results demonstrate that the proposed CDBN outperforms comparative models, achieving an F1 score of 0.9258. These results indicate that the architecture effectively overcomes the challenges of lesion classification in retinal images, offering a robust tool for clinical application and paving the way for advanced clinical decision support systems in diabetic retinopathy management.

## 1. Introduction

Macular edema (ME) is one of the most common symptoms associated with the pathophysiology of diabetes mellitus (DM) [1]. This metabolic disorder is mainly characterized by the presence of hyperglycemia, resulting from a deficiency in the production and/or action of insulin, a hormone generated by the pancreas [2]. The pathophysiology of diabetes mellitus involves multiple stages that affect various systems of the human body.

**Figure 1:**
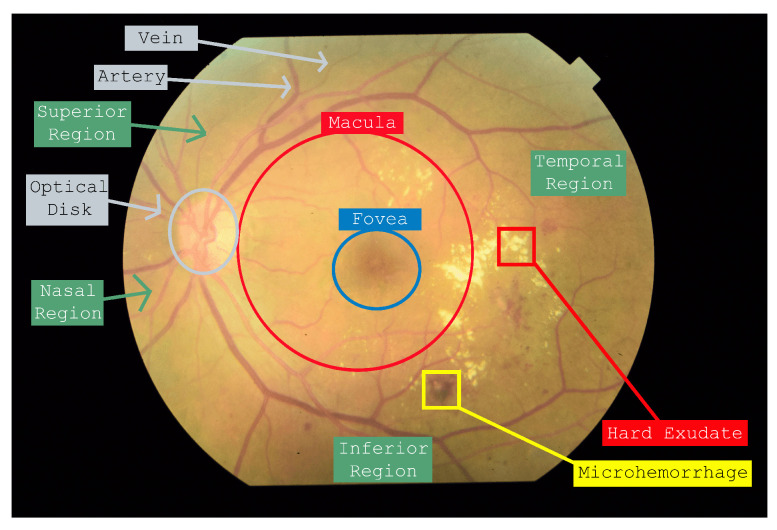
Labeling of different anatomy inside a retinal fundus image. All four regions (Temporal, Nasal, Superior and Inferior) are marked, as long as the fovea, macula, optic disc and a hard exudate and microhemorrhage example.

In particular, diabetes mellitus triggers processes that promote the production of free radicals, which play a crucial role in the deterioration of blood vessels [3]. The vascular damage can have significant effects, especially in the retina, favoring the development of diabetic retinopathy (DR) [4]. Within this context, ME emerges as a key manifestation that allows to evaluate the progression of DM in the patient [5]. Macular edema is characterized by the thickening of the macula, specifically in an area spanning two disc diameters from the fovea. It commonly manifests as accumulations of lipids, proteins and blood in the extracellular space of the retina, particularly in the macular region, located in the temporal region of the retina. The temporal region, as illustrated in Figure 1, is the area of the retina located closest to the temple. It lies between the superior and inferior regions and is opposite to the nasal region, which aligns with the direction of the nose and the optic disc. This region plays a critical role in the development of fine vision, making it particularly significant in the context of ocular health. The presence of abnormalities or injuries in the temporal region, such as those associated with ME, is of special importance. Detecting and addressing these issues early is crucial not only to prevent partial or total vision loss in patients but also to ensure proper monitoring and management from the initial stages of hyperglycemia through to the eventual development of DR. This makes the temporal region a key focus for both diagnosis and ongoing care in diabetic patients. Therefore, accurate and timely detection of EM is essential not only for optimal patient outcomes but also for improving overall clinical management and reducing the broader healthcare burden.

Macular edema has a wide variety of classifications, mainly depending on its clinical features defined by the detection method used, such as fundus fluorescein angiography, optical coherence tomography and retinal fundus images (RFI) [6] among others. Some types of edema can be stated as but not limited to diffuse edema, focal edema, clinically significant macular edema, vasogenic edema, edema when resulting from a rupture in the external blood-retinal barrier, tractional edema and ischemic maculopathy. The present work centers around two of the most common types of edema according to and defined by the High Specialities Medical Unit (UMAE) from the Mexican Institute of Social Security (IMSS), which are hard exudates and microhemorrhages (small areas of bleeding located within the layers of the retina).

Detecting and identifying different types of ME can be an issue since its diagnosis usually implicates taking a spatial context within the RFI [7], so that the position of the edema within the whole image and a neighborhood is usually the physician’s main tool. Other factors are taken into account, such as the patient’s medical history and family history. The development of specialists in the field capable of performing timely detection is crucial for patient health. However, this task can be hindered by various reasons. Among them is the high level of expertise required to correctly interpret data, due to the subtle or less evident ways in which ME presents itself in detection methods, as well as to properly distinguish between artifacts and real pathological findings. Additionally, extensive practical experience is required, as there are different classifications of ME, and it is necessary to have tailored practical knowledge for each geographic region and medical practice. This involves having labeled data according to specific needs, which adds a significant challenge for resident physicians when seeking learning materials. Typically, this level of expertise is achieved after years of practice and feedback from other experts. The availability of labeled data is limited.

Among the methods that rely on labeled data, current computational approaches primarily use Convolutional Neural Network (CNN)-based architectures for segmenting lesions like microaneurysms, exudates, hemorrhages, and other structures in RFI [8,9,10,11]. Preprocessing is a critical step, enhancing color normalization and reducing noise. Some approaches employ multi-stage CNNs to classify each pixel as either a lesion or non-lesion, achieving considerable accuracy in certain classes but facing challenges in processing time. Other CNN-based methods have been proposed to address the scarcity of labeled data, such as transfer learning [12], Glowworm Swarm Optimization for parameter tuning [13], and the integration of filters with traditional segmentation techniques like the Hough transform and Gabor filters [14,15]. This study introduces a method based on Convolutional Deep Belief Networks (CDBNs) for the classification of ME in cases of hard exudates and microhemorrhages, with focus on developing robust models trained on a RFI dataset characterized by noise, artifacts, and the coexistence of diverse anatomical structures. Unlike traditional CNNs, CDBNs leverage a hierarchical, layer-wise learning architecture to extract high-level features while preserving the spatial relationships inherent in medical images and specially in the diagnosis and identification of ME.

The hierarchical approach not only enhances its ability to discern subtle pathological patterns—such as the texture variations between exudates and microhemorrhages—but also inherently mitigates overfitting by learning generalized representations through unsupervised pre-training. This pre-training phase allows the CDBNs to capture intrinsic features, making them less dependent on large annotated datasets and more adaptable to the inherent variability found in real-world retinal images, where artifacts or overlapping anatomical components can obscure critical details. The proposed CDBN framework thus addresses these limitations by integrating multi-scale feature learning with probabilistic modeling, offering a more reliable solution for clinical applications where precision and adaptability to dataset imperfections are paramount. This paper is organized as follows: Section 2 introduces the specialist-provided, labeled database along with the employed methods. Section 3 presents the proposed method, while Section 4 details the training process, results, and a discussion of the method’s performance. Finally, Section 5 offers concluding remarks.

## 2. Materials and Methods

This section presents the main attributes of the dataset used, along with the key challenges it poses in practical applications. It then delineates the core principles underpinning the proposed model, highlighting the features that distinguish it from other state-of-the-art approaches.

### 2.1. Database of Macular Edema

**Figure 2:**
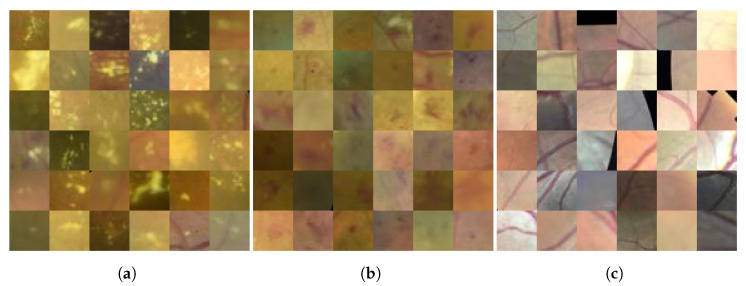
Samples for each macular edema class of the database: (**a**) patches containing hard exudates, (**b**) shows those with microhemorrhages, and column (**c**) includes healthy patches.

Manually hand-labeled MTA annotations have been created for this work by an expert ophthalmologist from the Ophthalmology Department of the Mexican Social Security Institute (IMSS) T1-León. The dataset comprises 130 RGB images showing ME and 100 images from healthy subjects, each with dimensions of 400×600 pixels. In the images positive for ME, 32×32-pixel boxes were used to mark the presence of two types of ME: hard exudates and microhemorrhages. In addition, 700 random patches were extracted from the regions of interest in healthy patient images, yielding a total of 755 patches for hard exudates and 718 for microhemorrhages. As shown in Figure 2, the different classes share certain characteristics such as color and intensity, whereas their morphological features are not always well defined. In fact, they can even contain anatomical structures such as veins and arteries within the same image. From these examples, one can observe the various color tones and subtle visual cues that make the classification process challenging. The overlapping characteristics among the classes underscore the need for robust methods capable of discriminating finer morphological details in ME diagnosis.

**Figure 3:**
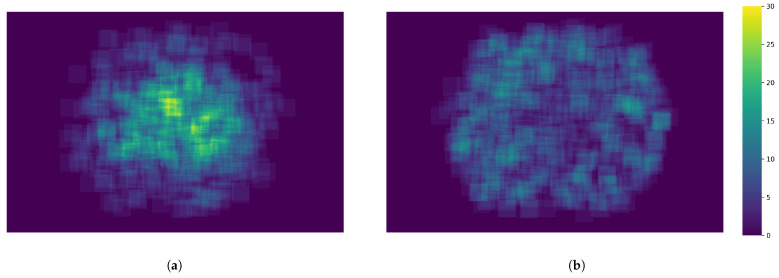
Macular edema database locations heatmap. Labels are denoted in a 32×32 pixel box indicating the existence of the type inside the box. (**a**) Locations for hard exudates; (**b**) Locations for microhemorrhages.

Furthermore, Figure 3 highlights the spatial distribution of these types of edema throughout the RFI. In particular, hard exudates tend to be more densely clustered at the center of the retina, whereas microhemorrhages are often located closer to the inner border. These distinct positional patterns suggest a physiological basis for each lesion type, reinforcing the importance of precise annotation strategies when building diagnostic models. A fundamental aspect in diagnosing the type of edema in fundus images is its location; however, this critical spatial information can be substantially lost when focusing exclusively on small patches. Moreover, the coexistence of multiple edema types within the same region further complicates the diagnosis, as it requires algorithms capable of discerning overlapping pathological signs.

### 2.2. Methods

#### 2.2.1. Restricted Boltzmann Machine

A Restricted Boltzmann Machine (RBM) is an unsupervised machine learning model that assumes independence among the data variables, belonging to the group of probabilistic models [16]. Commonly used to reduce the dimensional size of the data representation by learning the probabilistic distribution of the input data set, it is composed of two main layers: the visible layer responsible for receiving the input data and the hidden layer responsible for creating the low-level representation. For a low-level representation, it is of great interest to find a system configuration where the considerable use of neurons or units in the visible layer is lower. Taking the sum of the number of units in both layers together with their biases and weights as an indicative factor of this state, this translates into minimizing the total system value, also called energy [17]. This is analogous to finding the equilibrium state since the overall probability of the system being in the lower energy state follows a Boltzmann distribution making such states more likely. For *n* visible units *v* and *m* hidden units *h*, the energy function *E* is defined as

(1) $$E\left(\left\{v_{1},\ldots ,v_{n}\right\},\left\{h_{1},\ldots ,h_{m}\right\}\right)=\underset{\mathrm{visible}\;\mathrm{layer}}{\underbrace{-\sum_{j=1}^{m}b_{j}v_{j}}}-\underset{\mathrm{hidden}\;\mathrm{layer}}{\underbrace{\sum_{i=1}^{n}c_{i}h_{i}}}-\underset{\mathrm{weights}}{\underbrace{\sum_{i=1}^{n}\sum_{j=1}^{m}w_{ij}h_{i}v_{j}}}\;\;,$$

where *b* and *c* are the biases for the visible and hidden layers, respectively, and *w* are the weights between layers. The probability, and later importance to a more accurate representation of the input data, that a unit of the visible layer has when activated with respect to the hidden layer and, conversely, is defined as an independent Bernoulli random variable such that:

(2) $$\begin{array}{cc} P(h_{j}=1|\left\{v_{1},\ldots ,v_{n}\right\}) & =\sigma \left(\sum_{j}w_{ij}v_{j}+b_{j}\right),\\ P(v_{j}=1|\left\{h_{1},\ldots ,h_{m}\right\}) & =\sigma \left(\sum_{i}w_{ij}h_{i}+c_{i}\right),\\ \mathrm{where}\;\;\sigma (x) & ={\left(1+e^{-x}\right)}^{-1}. \end{array}$$

The previous probability is the main tool for the model to find an appropriate configuration. Minimizing this likelihood with respect to each bias and each unit weight represents a computational task with exponential complexity of the order of $2^{\min(n,m)}$; therefore, a common strategy is to employ a Gibbs Sampling method known as Contrastive Divergence, which approximates the gradient of the log-likelihood and updates the parameters accordingly. Due to its structure, an RBM, as described above, does not preserve the spatial information of the data, as it omits details about the particular structures present in the data. In contrast, a Boltzmann Machine does account for the dependence between variables, establishing relationships among each one and all the others [18]; however, these connections do not consider local topological features, which is especially important when working with images. Considering the above information and in view of the need to overcome the limitations in preserving the spatial structure, the Convolutional Restricted Boltzmann Machine (CRBM) proposal arises.

#### 2.2.2. Convolutional Restricted Boltzmann Machine

Building on this motivation, the CRBM leverages localized filtering and weight sharing to process images efficiently [19]. This design enables the model to capture relevant patterns without incurring the computational overhead of fully connected structures. For an input image at the visible layer, let each unit v(i,j) such that

(3) v(i,j)∈{0,1},i={1,…,I},j={1,…,J},

denote the binary state of the visible unit at the location (i,j). Instead of a single hidden layer vector, the CRBM uses *K* hidden feature maps and each feature map k={1,…,K}:

(4) $$h_{k}\left(i,j\right)\in \left\{0,1\right\},\;i=\left\{1,\ldots ,I^{\prime }\right\},\;j=\left\{1,\ldots ,J^{\prime }\right\},$$

is the binary state of the hidden unit at location (i,j) in the *k*th feature map. The dimensions $I^{\prime }$ and $J^{\prime }$ depend only on the size of the convolution filters and the boundary conditions used. Each hidden feature map is connected to the visible layer through a convolution with a kernel $w_{k}$ of size r×r. The weight filter $w_{k}$ is shared over all spatial locations of the corresponding feature map, which drastically reduces the number of free parameters compared to a fully connected RBM. The biases are also shared spatially. In particular, let $b_{k}$ denote the bias for the *k*th hidden feature map and *c* denote the bias for the visible units. With this set, in analogy with the standard RBM, the joint configuration {v,h} is assigned an energy function defined as

(5) $$E\left(v,\left\{h_{k}\right\}\right)=-\sum_{k=1}^{K}\sum_{i,j}h_{k}\left(i,j\right)\left[\left(w_{k}*v\right)\left(i,j\right)+b_{k}\right]-c\sum_{i,j}v\left(i,j\right),$$

where the convolution operator $\left(w_{k}*v\right)\left(i,j\right)$ is defined as

(6) $$\left(w_{k}*v\right)\left(i,j\right)=\sum_{p=0}^{r-1}\sum_{q=0}^{r-1}w_{k}\left(p,q\right)\;v\left(i+p,j+q\right).$$

Here the expression shows that each hidden unit $h_{k}\left(i,j\right)$ interacts only with a local patch of the visible layer, with the weights in that patch determined by the filter $w_{k}$. As in the standard RBM, the units in the CRBM are modeled as independent Bernoulli random variables when conditioned on the state of the opposite layer. For the hidden layer activation, given the visible image *v*, the probability that a hidden unit in the *k*th feature map at location (i,j) is active is given by

(7) $$P\left(h_{k}\left(i,j\right)=1|v\right)=\sigma \left(\left(w_{k}*v\right)\left(i,j\right)+b_{k}\right),$$

which implies that each hidden unit activation depends only on the weighted sum of the visible units in its receptive field plus the shared bias $b_{k}$. Conversely, given the set of hidden feature maps $\left\{h_{k}\right\}$, the probability that a visible unit at location (i,j) is active is computed by pooling the contributions from all feature maps, that can be expressed as

(8) $$P\left(v\left(i,j\right)=1|\left\{h_{k}\right\}\right)=\sigma \left(\sum_{k=1}^{K}\left({\tilde{w}}_{k}*h_{k}\right)\left(i,j\right)+c\right),$$

where ${\tilde{w}}_{k}$ denotes the flipped version of $w_{k}$ along both spatial dimensions, i.e.,

(9) $${\tilde{w}}_{k}\left(p,q\right)=w_{k}\left(r-1-p,\;r-1-q\right),$$

and the convolution is defined analogously:

(10) $$\left({\tilde{w}}_{k}*h_{k}\right)\left(i,j\right)=\sum_{p=0}^{r-1}\sum_{q=0}^{r-1}{\tilde{w}}_{k}\left(p,q\right)\;h_{k}\left(i+p,j+q\right).$$

This reconstruction step aggregates the back-projected contributions from all hidden feature maps to determine the activation probability of each visible unit. Building upon the capacity of the CRBM to capture local topological features, it becomes essential to incorporate a mechanism that not only reduces the dimensionality of the hidden representations but also enhances their invariance to small translations. Motivated by this need, probabilistic max pooling is introduced to aggregate the activations within local regions of each feature map into a more compact form. In contrast to a deterministic selection that simply picks the maximum value, probabilistic max pooling employs a stochastic approach to decide which activation within a pooling region is most significant, thereby preserving the probabilistic nature of the overall model. Concretely, consider a pooling region P within a given hidden feature map. Let ${\left\{h_{k}\left(i,j\right)\right\}}_{(i,j)\in P}$ denote the activations of the hidden units in this region for the *k*th feature map. The pooling operation is then defined by assigning a probability that a pooled unit is active according to the relative strengths of the hidden activations, expressed as

(11) $$P\left(h_{k}^{P}=1|{\left\{h_{k}\left(i,j\right)\right\}}_{(i,j)\in P}\right)=\frac{\sum_{(i,j)\in P}\exp\left(h_{k}\left(i,j\right)\right)}{1+\sum_{(i,j)\in P}\exp\left(h_{k}\left(i,j\right)\right)}\;,$$

where the additional unit term in the denominator accounts for the possibility that none of the units in the pooling region become active. The formulation enforces a competitive mechanism among the hidden units, ensuring that, with high probability, only one unit per pooling region dominates the pooled representation. By leveraging probabilistic max pooling, redundancy and computational complexity are reduced while facilitating the extraction of more robust, invariant features. When combined with the CRBM, the pooling mechanism lays the groundwork for constructing a Convolutional Deep Belief Network (CDBN), wherein successive layers can build upon these refined representations to capture increasingly abstract characteristics of the input data.

## 3. Proposed Convolutional Deep Belief Network

**Figure 4:**
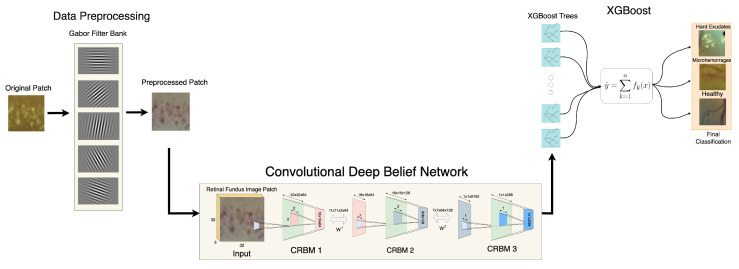
Proposed Convolutional Deep Belief Network. From left to right in: The original image patch undergoes preprocessing, where the Gabor filter bank is applied to enhance features (top-left block). The resulting preprocessed patch is then fed into the CDBN (upper center block), depicted with three CRBM layers that successively extract increasingly complex characteristics. Once the high-level features (a 256-dimensional vector) are obtained from the final CRBM layer, they serve as input to the XGBoost classifier (bottom-right block). Here, 100 decision trees are sequentially fitted to refine the model, culminating in the final classification of retinal pathologies—such as hard exudates, microhemorrhages, or healthy patches—based on the predicted label.

In the present work, a novel method based on a Convolutional Deep Belief Network is introduced consisting of three main stages: Preprocessing, Unsupervised Training, and Classification. As illustrated in Figure 4, the first stage involves feature enhancement using a Gabor filter applied independently to each color channel. The second stage focuses on the unsupervised training of the preprocessed dataset to extract relevant features, specifically high-level features obtained from the last layer. This is carried out based on the architecture proposed by Lee et al. [19], which is composed of two convolutional layers. The choice of high-level features follows from the nature of the model, which is capable of learning more discriminative representations in deeper layers, ultimately extracting a 256-dimensional feature vector. Finally, in the third stage, an XGBoost [20] model is trained. XGBoost is a boosting algorithm that optimizes the loss function in function space (decision trees) by means of a second-order approximation. Introducing each new tree in this context adds it sequentially to correct errors of the previous model, for which a total of 100 trees was selected.

Due to the CDBN sensitivity to both its training parameters and the intrinsic characteristics of the dataset, metaheuristics were employed to identify the parameter set that yields optimal performance. Adopting an evolutionary-based approach, three state-of-the-art algorithms were considered: the Real-Coded Genetic Algorithm, the Boltzmann Univariate Marginal Distribution Algorithm (BUMDA) [21], and the Univariate Marginal Distribution Algorithm (UMDA) [22]. These algorithms were tasked with finding the parameter combination that maximizes the proposed CDBN in terms of the F1 Score metric.

The proposed CDBN stands out among existing state-of-the-art approaches primarily due to its reliance on high-level features extracted from the final layer of the CDBN. It is noteworthy that the employed dataset comprises ME retinal images with significant levels of noise, making these images highly similar in their low-level features. By focusing on higher-level representation, the method alleviates the risk of confusion introduced by noise, since such representations capture more abstract patterns that are distinct enough to differentiate pathological findings from healthy tissue. Within the unsupervised training phase described, the deeper layers of the network learn feature maps specifically tailored to these noisy retinal images. This emphasis on high-level features is especially advantageous for ME detection, wherein slight variations in color and texture—common in lower-level representations—can be overshadowed by the presence of noise. By selecting only the final layer’s output for subsequent XGBoost classification, the proposed method confines the training process to the most discriminative characteristics. This approach, in turn, enhances the robustness of the classification, despite the intrinsic challenges posed by the RFI. Thus, as shown in Figure 4, the three-step architecture—consisting of Preprocessing, Unsupervised Training, and Classification—provides a comprehensive pipeline that addresses the specific demands of ME image analysis. In comparison to conventional techniques that often rely on both low- and mid-level features, the strategy leverages only the essential high-level representations, leading to improved accuracy and stability in the face of noisy inputs.

## 4. Results and Discussion

All the experiments were conducted using a system equipped with two NVIDIA T4 GPUs, 29 GB of RAM. The implementation was done in Python 3.11.8 using the PyTorch 2.4.0 framework. For the pre-processing and unsupervised training stages, it is required to establish a total of 11 corresponding parameters. Specifically, the parameters σ, θ, λ, ψ, γ, and *k* pertain to the Gabor filter applied during pre-processing, while the parameters $p_{1}$ and $p_{2}$ for max pooling probabilities, $\alpha_{1}$, $\alpha_{2}$ and $\alpha_{3}$ for the learning rates, are associated with the unsupervised training phase. Finding the optimal parameters poses significant challenges, as the CDBN is a highly sensitive system with respect to its parameter configuration, compounded by a database with unknown discriminative characteristics.

**Figure 5:**
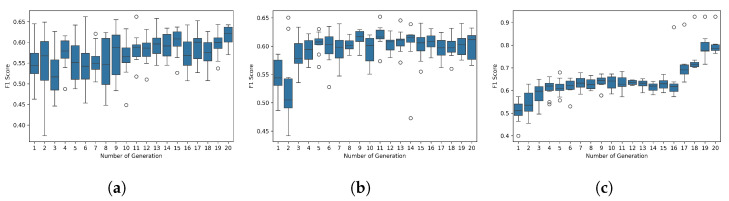
Boxplots of F1 Scores across 20 generations for three evolutionary algorithms applied to parameter optimization of an unsupervised model and a Gabor filter: (**a**) BUMDA, (**b**) UMDA, and (**c**) Real-Coded Genetic Algorithm. Each boxplot summarizes the F1 distribution for the entire population at each generation. BUMDA (**a**) exhibits broader variability in early generations but converges toward higher F1 values after approximately the 10th generation. UMDA (**b**) shows a more consistent upward trend in F1 over the generations, with progressively narrower distributions. The Real-Coded Genetic Algorithm (**c**) demonstrates a steady increase in median F1, although the distributions remain relatively compact compared to the other methods.

The adopted approach consisted of determining these 11 parameters by means of a search using three evolutionary algorithms: the Real-Coded Genetic Algorithm, the Boltzmann Univariate Marginal Distribution Algorithm, and the Univariate Marginal Distribution Algorithm. The search was conducted over 20 generations with a population size of 10, and the F1 Score was employed as the metric to be maximized. The search ranges were defined as follows: σ∈[100.0,200.0], θ∈[0,π], λ∈[8.0,16.0], ψ∈[0,2π], γ∈[0.5,1.0], k∈[45,180], $p_{1},p_{2}\in \left[0,1.0\right]$, $\alpha_{1},\alpha_{2},\alpha_{3}\in \left[10^{-9},10^{-5}\right]$. Figure 5 presents a boxplot illustrating the distribution of the population in each generation for each algorithm. This representation notably emphasizes the considerable variety of results obtained, thereby highlighting the richness of the exploration space.

**Figure 6:**
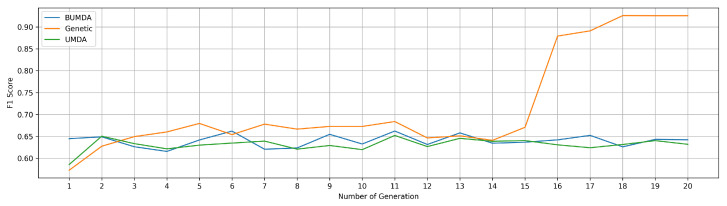
Elite individuals across generations. Due to the sensitivity of the model to the hyperparameters and the change of features within the database introduced by the gabor filter, the model tends to have premature convergence regardless of the evolutionary algorithm used.

The best parameters correspond to the experiment with σ=197.8324, θ=0.4148, λ=14.6398, ψ=0.4470, γ=0.9831, k=71.3538, $p_{1}=0.4227$, $p_{2}=0.9839$, $\alpha_{1}=3.0260\times 10^{-6}$, $\alpha_{2}=4.1494\times 10^{-6}$, $\alpha_{3}=3.4456\times 10^{-7}$ as determined by the Real-Coded Genetic Algorithm. Figure 6 shows the evolution of the elite individual in each generation, illustrating an initial behavior common to all algorithms and emphasizing the Real-Coded Genetic Algorithm capacity to adequately diversify the proposals in accordance with the problem. It is important to highlight that the usage of the Gabor filter substantially enhances the proposed CDBN: without this stage the network attains an accuracy of 0.8465 and an F1 score of 0.8424 with 68 misclassifications. Its inclusion raises accuracy to 0.9264, F1 to 0.9258, and lowers the errors to 33, evidencing the filters decisive contribution.

For comparative purposes, an extensive set of state-of-the-art CNN as well as several variants of Convolutional Deep Belief Networks have been rigorously evaluated. The CNN-based models, enumerated as (1) through (10), are categorized into four distinct groups based on factors such as network depth, parameter count, and innovative architectural features:

- **Group 1 (Residual Networks):**
  – **ResNet50 (1)** and **ResNet101 (2)** belong to the family of residual networks incorporating skip connections to alleviate the vanishing gradient problem and facilitate the training of deeper architectures. ResNet50 employs 50 layers, achieving a judicious balance between depth and computational efficiency, while ResNet101, with its 101 layers, provides enhanced representational capacity.
- **Group 2 (Classical and Modern Convolutional Approaches):**
  – **DenseNet121 (3)** leverages dense connectivity whereby each layer receives the aggregated feature maps of all preceding layers, thereby promoting improved gradient flow and efficient feature reuse.
  – **VGG16 (4)** represents a classical CNN architecture characterized by a straightforward sequential arrangement of convolutional layers with small 3×3 filters; its simplicity remains competitive in performance.
  – **ConvNeXt Small (6)** revisits traditional convolutional architectures by integrating modern design principles inspired by transformer-based models, refining conventional blocks while maintaining efficiency.
- **Group 3 (Inception-Based Architectures):**
  – **InceptionV3 (5)** utilizes parallel convolutional paths with filters of various sizes within the so-called “Inception modules”, thereby efficiently capturing multi-scale features.
  – **InceptionResNetV2 (7)** synergistically combines the Inception framework with residual connections, further enhancing gradient propagation and reducing the complexity of the training process.
- **Group 4 (Depthwise Separable Convolution and Neural Architecture Search):**
  – **Xception (8)** decomposes standard convolutional operations into depthwise and pointwise convolutions, thus increasing efficiency while preserving the depth of representation.
  – **NASNet Large (9)** is derived from a Neural Architecture Search strategy, which autonomously discovers an optimized network topology tailored for superior accuracy in large-scale vision tasks.
  – **MobileNet (10)** similarly employs depthwise separable convolutions, albeit with a strong emphasis on lightweight design, rendering it particularly well-suited for computationally constrained environments.

**Figure 7:**
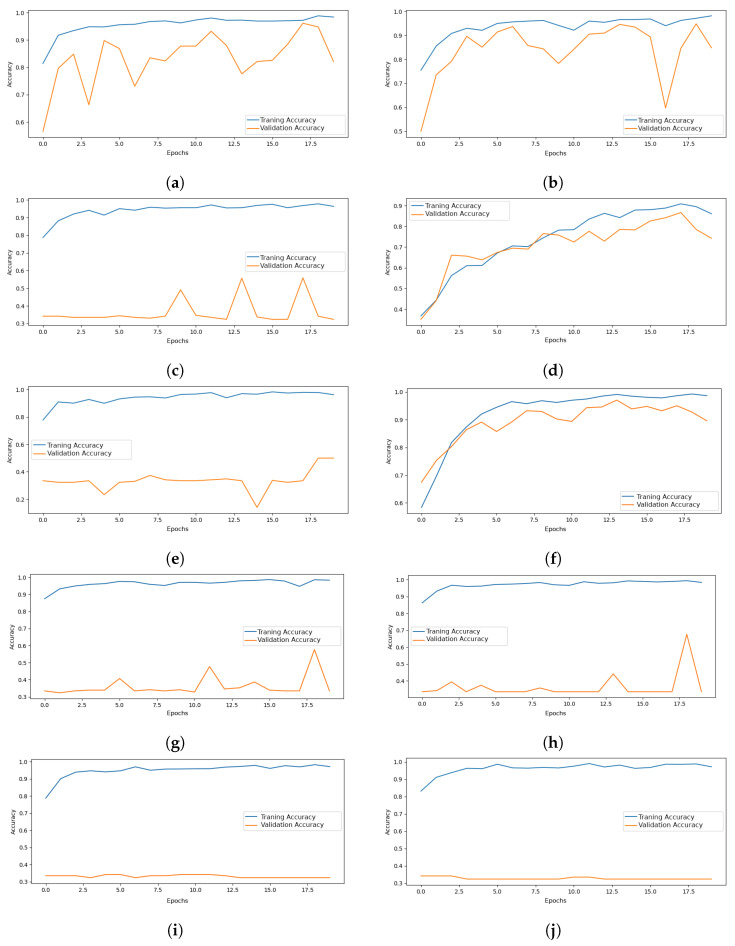
Performance of comparative architectures in terms of accuracy: (**a**) ResNet50, (**b**) ResNet101, (**c**) DenseNet121, (**d**) VGG16, (**e**) InceptionV3, (**f**) ConvNeXt Small, (**g**) InceptionResNetV2, (**h**) Xception, (**i**) NASNet Large, and (**j**) MobileNet.

Among the evaluated models, the Convolutional Deep Belief Network framework, as introduced by [19], is of particular relevance. In this approach, only the features extracted from the first and second layers are employed for classification. This design capitalizes on the hierarchical representations captured in the early stages of the network, which are then directly utilized to achieve effective classification performance. Although these CNN-based models offer diverse disadvantages, as evidenced in Figure 7, it is noteworthy that a prevalence of overfitting models can be discerned. Specifically, InceptionResNetV2, Xception, NASNet Large, MobileNet, InceptionV3, and DenseNet121 exhibit a marked tendency to converge prematurely to local minima from the onset of training on the RFI database.

**Figure 8:**
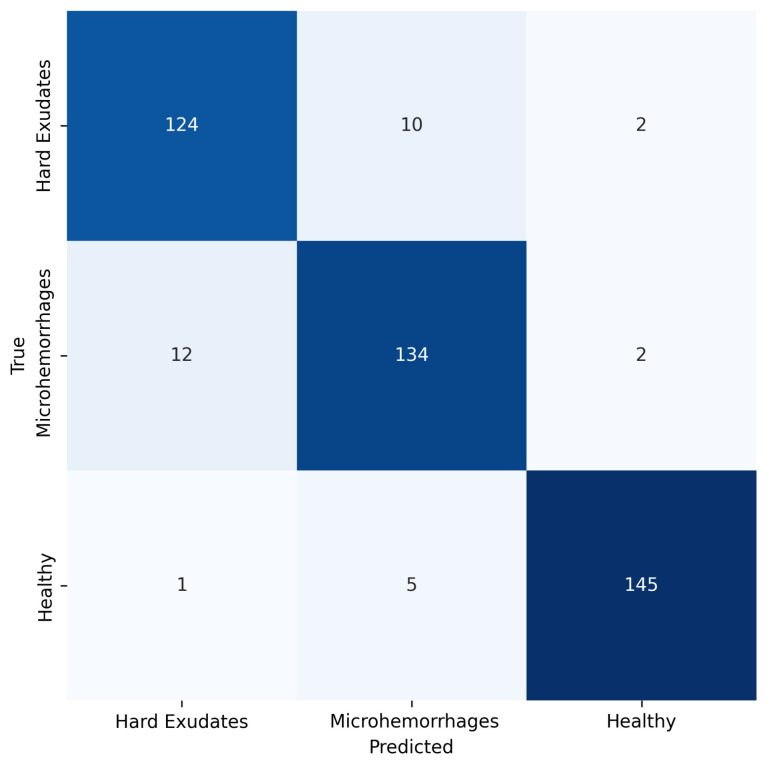
Confusion matrix of the proposed CDBN classifying hard exudates, microhemorrhages, and healthy samples. Misclassifications are relatively small and occur mostly between hard exudates and microhemorrhages, suggesting that these two pathologies may share similar features. The minimal confusion with the healthy class further highlights the model robustness in distinguishing diseased from non-diseased samples.

Finally, Table 1 consolidates the performance metrics of all models. Notably, the proposed CDBN surpasses existing CNN-based approaches and earlier CDBN variants, with a training time of 245 s. Its superior accuracy, F1 score, recall, and precision highlight the efficacy of combining deep feature extraction with an ensemble-based classifier to address the challenges of ME detection, by employing a configuration that relies on high-level features extracted from the last layer. In Figure 8, the confusion matrix of the proposed CDBN is presented, where it can be noted that the model is able to make a fairly good distinction between the healthy class and the other two, suggesting that there is a strong similarity between the characteristics of the edema types.

**Figure 9:**
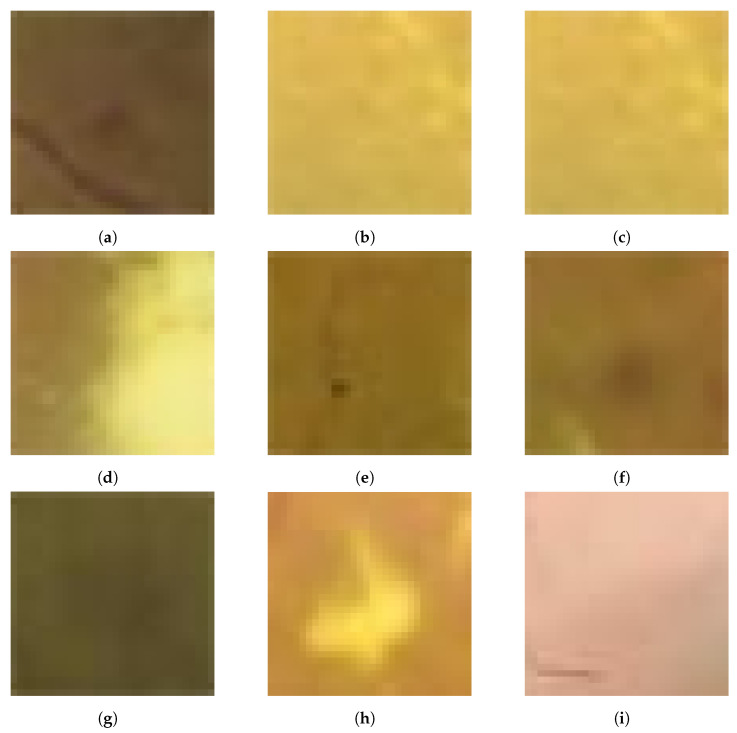
Subset of true positive classificated patches: (**a**) microhemorrhages; (**b**) healthy; (**c**) hard exudates; (**d**) hard exudates; (**e**) microhemorrhages; (**f**) microhemorrhages; (**g**) microhemorrhages; (**h**) hard exudates; (**i**) healthy.

**Figure 10:**
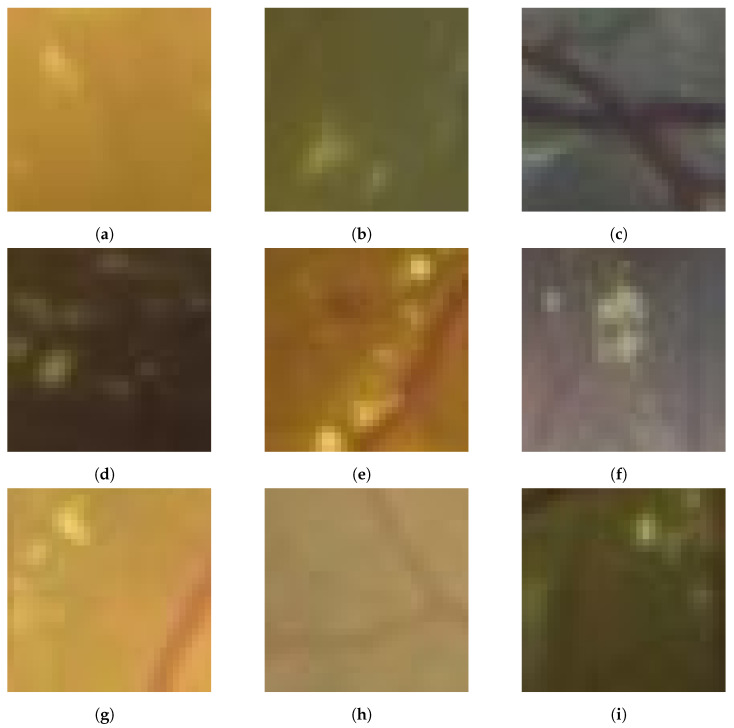
Subset of false positive classificated patches. (**a**) Label: hard exudates. Predicted label: microhemorrhages; (**b**) Label: hard exudates. Predicted label: microhemorrhages; (**c**) Label: healthy. Predicted label: microhemorrhages; (**d**) Label: hard exudates. Predicted label: microhemorrhages; (**e**) Label: hard exudates. Predicted label: microhemorrhages; (**f**) Label: hard exudates. Predicted label: healthy; (**g**) Label: hard exudates. Predicted label: microhemorrhages; (**h**) Label: healthy. Predicted label: hard exudates; (**i**) Label: hard exudates. Predicted label: microhemorrhages.

In order to visualize the ME classification results, correctly classified and incorrectly classified images are presented in Figure 9 and Figure 10, respectively. Among the correctly classified images, it is evident that there is a notable robustness with regard to the microhemorrhages and healthy classes, as the model can accurately distinguish small spots within adjacent noise—noise whose intensity is comparable to that of the hard exudates class. Furthermore, the model is capable of discerning various structures for the microhemorrhages class without being constrained to a particular subgroup. In the set of incorrectly classified images, the model exhibits a tendency to confuse the hard exudates class with microhemorrhages. This may be attributed to the fact that several samples share features of these two classes and, to a lesser extent, the healthy and microhemorrhages classes as well, owing to the rupture of veins adjacent to other anatomical structures that do not necessarily signal the presence of the class. Additional challenges for the model include intensity variations arising from factors such as the anatomical configuration of the eye and the flash intensity employed during image capture, as well as natural tonal variations in the macula—which collectively suggest that the model may erroneously interpret these as instances of microhemorrhage.

The core contribution of the proposed method lies in exclusively leveraging high-level features extracted from the final CDBN layer, a strategy critical to overcoming the strong resemblance of low-level characteristics prevalent in noisy images or closely related classes. The preprocessing stage employs Gabor filters, while the subsequent parameter tuning through metaheuristics—namely evolutionary algorithms—provides an effective means to explore the high-dimensional search space.

A notable element of this proposal is its ability to preserve spatially significant information in the macular region, which is vital given the potential overlap between lesions and anatomical structures. Employing convolutional layers within the CRBM architecture, together with probabilistic pooling, enables the capture of abstract and robust patterns that yield enhanced classification stability and accuracy. Comparisons with state-of-the-art CNN models show that the CDBN-based strategy surpasses conventional methods in metrics such as the F1 Score, underscoring the importance of focusing on high-level feature representations for medical image pathology detection.

Nonetheless, Figure 8 reveals a slight inclination to misclassify hard exudates and microhemorrhages, suggesting shared attributes between these two lesion types. This finding indicates that refining segmentation or incorporating, for instance, additional attention mechanisms could lead to a more precise differentiation. In addition, despite the efficiency of the hyperparameter optimization process, results highlight the sensitivity of the model to critical parameter settings, which can lead to premature convergence under conditions of extreme data variability.

In summary, combining a robust CDBN architecture with an XGBoost-based classification method offers a powerful framework for ME classification. Its ability to extract discriminative features in highly variable and noisy environments constitutes a significant advance over the state of the art.

## 5. Conclusions

The present study introduces a novel Convolutional Deep Belief Network architecture whose function is the automatic ME classification in RFI. The proposed method is structured in three distinct stages: preprocessing, unsupervised learning, and classification, with each stage rigorously integrated within a metaheuristic-driven parameter optimization framework. Thus, in the preprocessing stage, a Gabor filter is employed to highlight features that are significant for diagnosis, with the Real-Coding Genetic Algorithm being the tool used to determine the optimal parameters, both for the filtering process and for the subsequent CDBN. This accurate calibration not only improves feature extraction, but also mitigates the challenges posed by noisy images and high similarity of low-level features. However, a central contribution of this work lies in its exclusive reliance on high-level feature representations extracted from the final CDBN layer. This approach allows the model to capture abstract and robust patterns that are critical for differentiating between hard exudates, microhemorrhages, and healthy tissue, even in the presence of considerable variability and noise. Also, the integration of convolution and probabilistic pooling layers within the CRBM framework further strengthens the spatial fidelity of features in the macular region, thus contributing to greater stability and accuracy in classification. Importantly, empirical evaluations demonstrate that the proposed CDBN achieves an F1 score above 0.92, accompanied by high levels of precision, accuracy, and recall, outperforming several state-of-the-art models. It is important to acknowledge the model marked sensitivity to the specific configuration of hyperparameters, affecting both the Gabor filtering stage and the learning dynamics of the CDBN. This sensitivity results in a rigorous optimization process, like the one conducted using evolutionary algorithms, to achieve optimal performance. Consequently, robust application of the model in different clinical settings might require careful recalibration to adapt to inherent variations within the data. Finally, the proposed CDBN represents a substantial advance in the field of medical image analysis for diabetic retinopathy. By combining robust unsupervised learning with an effective metaheuristic search strategy and an advanced classification framework, the proposed method not only addresses current diagnostic challenges, but also lays the foundation for future developments in clinical decision support systems. Thus, the methodological ability to extract high-level discriminative features and maintain spatially relevant information in complex and noisy environments provides a fundamental contribution to the advancement of automated retinal pathology detection. Future work will focus on enhancing this foundation by incorporating diverse methods such as attention mechanisms, which promise to exploit high-level discriminative features more effectively, and by exploring information fusion strategies or hybrid architectures. Such solutions combine learned representations at multiple levels to further refine the distinction among various edema types and other retinal pathologies sharing similar challenges. Once these improvements and integrations are in place, the method’s powerful discriminative capability and computational efficiency suggest that it could be effectively integrated into clinical decision-support systems. Consequently, it would enable earlier, more accurate detection of macular edema, thereby facilitating timely interventions and improving prognoses for diabetic patients—a direct contribution to preserving vision and alleviating healthcare burdens. 

## Figures and Tables

**Table 1:** Performance metrics of different models.

Model	Accuracy	F1 Score	Recall	Precision	Time (s)
MobileNetV3 [23]	0.3235	0.1581	0.3235	0.1046	$1.40034\times 10^{-2}$
DenseNet121 [24]	0.3235	0.1581	0.3235	0.1046	$4.88262\times 10^{-2}$
NASNetLarge [25]	0.3235	0.1581	0.3235	0.1046	$7.93386\times 10^{-2}$
Xception [26]	0.3348	0.1679	0.3348	0.1121	$1.23789\times 10^{-2}$
InceptionResNetV2 [27]	0.3348	0.1679	0.3348	0.1121	$4.85893\times 10^{-2}$
InceptionV3 [28]	0.5	0.4359	0.5	0.7816	$2.72754\times 10^{-2}$
MobileNetV3Large [29]	0.6199	0.5295	0.6199	0.7316	$1.89715\times 10^{-2}$
EfficientNetB7 [30]	0.6765	0.6702	0.6805	0.7641	$8.05643\times 10^{-2}$
VGG16 [31]	0.7420	0.7152	0.7420	0.7517	$4.73567\times 10^{-3}$
CDBN [19]	0.7720	0.7632	0.7666	0.7637	$2.71863\times 10^{-5}$
ResNet50 [32]	0.8212	0.8184	0.8212	0.8338	$2.33246\times 10^{-2}$
ResNet101 [32]	0.8484	0.8477	0.8484	0.8629	$4.73445\times 10^{-2}$
ConvNeXtSmall [33]	0.8959	0.8909	0.8959	0.9104	$2.46732\times 10^{-2}$
**Proposed CDBN**	**0.9264**	**0.9258**	**0.9258**	**0.9259**	$1.02753\times 10^{-4}$

## Data Availability

The original contributions presented in this study are included in the article. Further inquiries can be directed to the corresponding author.

## Abbreviations

The following abbreviations are used in this manuscript:

ME	Macular Edema
DR	Diabetic Retinopathy
BM	Boltzmann Machine
RBM	Restricted Boltzmann Machine
DBN	Deep Belief Network
CDBN	Convolutional Deep Belief Network
UMAE	High Specialities Medical Unit
RFI	Retinal Fundus Images
